# Genome Sequence of *Anoxybacillus geothermalis* Strain GSsed3, a Novel Thermophilic Endospore-Forming Species

**DOI:** 10.1128/genomeA.00575-15

**Published:** 2015-06-11

**Authors:** Sevasti Filippidou, Marion Jaussi, Thomas Junier, Tina Wunderlin, Ludovic Roussel-Delif, Nicole Jeanneret, Andrea Vieth-Hillebrand, Alexandra Vetter, Simona Regenspurg, Shannon L. Johnson, Kim McMurry, Cheryl D. Gleasner, Chien-Chi Lo, Paul Li, Momchilo Vuyisich, Patrick S. Chain, Pilar Junier

**Affiliations:** a Laboratory of Microbiology, Neuchâtel, Switzerland; b Vital-IT Group, Swiss Institute of Bioinformatics, Lausanne, Switzerland; c German Research Centre for Geosciences GFZ, Potsdam, Germany; d Los Alamos National Laboratory, Los Alamos, New Mexico, USA

## Abstract

*Anoxybacillus geothermalis* strain GSsed3 is an endospore-forming thermophilic bacterium isolated from filter deposits in a geothermal site. This novel species has a larger genome size (7.2 Mb) than that of any other *Anoxybacillus* species, and it possesses genes that support its phenotypic metabolic characterization and suggest an intriguing link to metals.

## GENOME ANNOUNCEMENT

From 2013 to date, 10 genome sequences of a total of 23 species that belong to the *Anoxybacillus* genus have been announced. GSsed3 (also known as ATCC BAA-2555) is the type strain of the novel species *Anoxybacillus geothermalis*, isolated in 2011 from above-ground filter deposits of the geothermal site in Gross Schönebeck, in the North German Basin (52°54′13.15″N 13°36′5.43″E) (S. Filippidou, M. Jaussi, N. Jeanneret, L. Roussel-Delif, T. Wunderlin, A. Vieth-Hillebrand, A. Vetter, P. Chain, S. Regenspurg, and P. Junier, unpublished data). All *Anoxybacillus* species are moderately thermophilic, and most of them have been isolated from geothermal sites. According to its 16S rRNA gene sequence, *A. geothermalis* strain GSsed3 is closely related to *Anoxybacillus rupiensis* (1), whose full genome sequence is not yet available. The genome of GSsed3 was sequenced and annotated, and its metabolic capabilities were revealed.

Genomic DNA was extracted from an overnight culture using the QIAamp DNA minikit (Qiagen GmbH, Germany). The draft genome of *A. geothermalis* strain GSsed3 was generated by the Los Alamos National Laboratory (LANL) Genome Science Group using Illumina (2) technology. For this genome, an Illumina short-insert paired-end library was constructed and sequenced, generating 21,691,466 reads totaling 2,191 Mbp. The Illumina draft data were assembled with Velvet version 1.2.08 (3). The estimated size of the genome is 7.2 Mb, and the final assembly is based on 1,330 Mbp of Illumina draft data, which provide 185× coverage of the genome. The genomic DNA G+C content is estimated to be 46.8 mol%. Genome annotation was performed using an Ergatis-based (4) workflow with minor manual curation and visualized with the Artemis genome browser and annotation tool (5).

The complete genome sequence contained 7,003 genes, 7 rRNAs (5S, 16S, and 23S), 72 tRNAs, and 2 noncoding RNAs (ncRNAs) predicted. This strain possesses a large genome compared to those of other *Anoxybacillus* species, whose genomes do not exceed 3.3 Mb. In addition to its size, the genome contains more than double the number of genes in other published genomes of this genus (6–12). Among the annotated genes, 143 genes encoding proteins related to sporulation and nine genes related to dipicolinic acid synthesis were found. GSsed3 was biochemically tested and found to hydrolyze urea and reduce nitrates to nitrites. Genome annotation supports these findings, since it revealed the presence of the loci *ureE* and *ureC* for urease activity (13) and two copies of a putative nitrate reductase. It assimilates mannitol as an alternative carbon source to glucose. Six proteins encoding mannitol transporters and dehydrogenases were found. Similarly, ribose ABC transporter permeases have been found, which explains the assimilation of ribose. Interestingly, this *Anoxybacillus* species is found to weakly assimilate xylan, and its genome contains genes for 1,4-β-xylanase, a typical characteristic of the genus *Geobacillus* (14) rarely observed in *Anoxybacillus* (Filippidou et al., unpublished data). Finally, binding proteins and transporters of copper, manganese, cadmium, iron, and zinc were found, as well as an arsenic resistance protein (ArsB). Copper oxidase and manganese catalase genes are also present.

### Nucleotide sequence accession numbers.

This whole-genome shotgun project has been deposited at DDBJ/EMBL/GenBank under the accession no. JYCG00000000. The version described here is JYCG00000000.1.
